# Serum markers of fibrosis, cardiovascular and all-cause mortality in hemodialysis patients: the AURORA trial

**DOI:** 10.1007/s00392-021-01898-9

**Published:** 2021-06-25

**Authors:** Madonna Salib, Sophie Girerd, Nicolas Girerd, Winfried März, Hubert Scharnagl, Ziad A. Massy, Céline Leroy, Kévin Duarte, Hallvard Holdaas, Alan G. Jardine, Roland E. Schmieder, Bengt Fellström, Natalia López-Andrés, Patrick Rossignol, Faiez Zannad

**Affiliations:** 1 Université de Lorraine, Inserm, Centre d’Investigations Cliniques-1433, and Inserm U1116, CHRU Nancy; F-CRIN INI-CRCT, Nancy, France; 2 Nephrology Department, University Hospital of Nancy, Vandoeuvre les Nancy, Nancy, France; 3 Medical Clinic V (Nephrology, Hypertensiology, Rheumatology, Endocrinology, Diabetology), Medical Faculty Mannheim, University of Heidelberg, Theodor-Kutzer-Ufer 1-3, 68167 Mannheim, Germany; 4 Clinical Institute of Medical and Chemical Laboratory Diagnostics, Medical University of Graz, 8036 Graz, Austria; 5 Division of Nephrology, Ambroise Paré University Hospital, APHP, Boulogne Billancourt/Paris, and INSERM U-1018, Centre de recherche en épidémiologie et santé des populations (CESP), Equipe 5, Paris-Saclay University (PSU) and University of Paris Ouest-Versailles-Saint-Quentin-en-Yvelines (UVSQ), FCRIN INI-CRCT, Villejuif, France; 6 Medical Department, Rikshospitalet, University of Oslo, Oslo, Norway; 7 Renal Research Group, British Heart Foundation Cardiovascular Research Centre, Institute of Cardiovascular and Medical Sciences, University of Glasgow, Glasgow, UK; 8 Department of Nephrology and Hypertension, University Hospital Erlangen, Erlangen, Germany; 9 Department of Nephrology, University Hospital, Uppsala, Sweden; 10 Navarrabiomed, Complejo Hospitalario de Navarra (CHN), Universidad Pública de Navarra (UPNA), IdiSNA, Pamplona, Spain

**Keywords:** Hemodialysis, Collagen, Fibrosis, Biomarker, Galectin-3, Cardiovascular diseases

## Abstract

**Background:**

Biomarkers of fibrosis are associated with outcome in several cardiovascular diseases. However, their relevance to chronic kidney disease and dialysis is uncertain, as it remains unclear how the kidneys and the dialysis procedure itself affect their elimination and degradation. We aimed to investigate the relationship of the blood levels of two markers associated with fibrosis: procollagen type I C-terminal pro-peptide (PICP) and galectin-3 (Gal-3) with mortality in dialysis patients.

**Methods:**

Procollagen type I C-terminal pro-peptide and galectin-3 were measured at baseline in 2773 patients enrolled in the AURORA trial, investigating the effect of rosuvastatin on cardiovascular outcomes, in patients on hemodialysis, and their interaction with CV death or all-cause mortality using survival models. The added prognostic value of these biomarkers was assessed by the net reclassification improvement (NRI).

**Results:**

The median follow-up period was 3.8 years. Blood concentrations of PICP and Gal-3 were significantly associated with CV death [adjusted HR per 1 SD = 1.11 (1.02–1.20) and SD = 1.20 (1.10–1.31), respectively] and all-cause mortality (all adjusted *p* < 0.001). PICP and Gal-3 had a synergistic effect with regard to CV death and all-cause mortality (interaction *p* = 0.04 and 0.01, respectively). Adding PICP, Gal-3 and their interaction on top of clinical and biological covariates, resulted in significantly improved prognostic accuracy NRI = 0.080 (0.019–0.143) for CV death.

**Conclusion:**

In dialysis patients, concomitant increase in PICP and Gal-3 concentrations are associated with higher rates of CV death. These results suggest that concomitantly raised PICP and Gal-3 may reflect an activated fibrogenesis relevant to risk stratification in dialysis, raising the hypothesis that anti-fibrotic therapy may be beneficial for cardiovascular protection in such patients.

**Graphic abstract:**

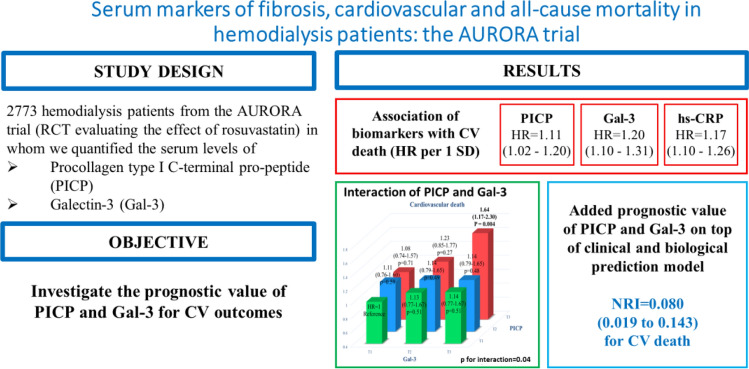

**Supplementary Information:**

The online version contains supplementary material available at 10.1007/s00392-021-01898-9.

## Introduction

Cardiovascular (CV) diseases are the leading cause of mortality in patients on dialysis. But for now, no therapeutic intervention was proven to improve CV outcomes [1, 2].

Several studies reported that patients with end-stage renal disease (ESRD) and undergoing hemodialysis (HD) have increased level of inflammation and oxidative stress, which are associated with a higher risk of CV death [3–6]. These mechanisms can lead to high collagen turnover resulting in fibrosis [7], which could be a therapeutic target.

In heart failure (HF), circulating biomarkers of myocardial fibrosis, in particular collagen turnover biomarkers, may be useful for predicting clinical risk or response to treatment [8, 9]. Collagen I and III are also identified as relevant biomarkers of vascular disease in chronic kidney disease (CKD), as they were associated with arterial stiffness [10, 11]. Nevertheless, in non-dialysis CKD patients, the interpretation of collagen biomarkers may be difficult as they may reflect the process of renal fibrosis, the decrease of the glomerular filtration rate (for biomarkers of low molecular weight), increased bone collagen turnover, as well as fibrosis in non-renal and non-cardiovascular tissue (i.e., liver or lungs) [12].

Serum procollagen type I C-terminal pro-peptide (PICP) was found to be correlated with total myocardial collagen volume fraction in patients with hypertensive heart disease. This would suggest that PICP is a key marker of the development of myocardial fibrosis [13, 14].

Galectin-3 (Gal-3) is a marker of inflammation and is involved in aldosterone-mediated fibrosis [15, 16]. According to guidelines [17], Gal-3 can be used for additional risk stratification in HF. Gal-3 acts as a profibrotic agent within the kidneys and therefore high plasma concentrations precede the development of CKD [15, 16]. Considering the negative association between Gal-3 and renal functions, its prognostic utility for CV disease in non-dialyzed CKD is controversial [18], but in dialysis, several studies reported an association between the concentration of Gal-3 and CV mortality [19–22].

In this framework, the investigation of PICP, a circulating collagen synthesis biomarker, in combination with Gal-3 could be a future strategy to identify patients at particularly high CV risk in hemodialysis, because these biomarkers may reflect active cardiovascular fibrogenesis.

We primarily aimed to evaluate the association of PICP and Gal-3 with adjudicated CV outcomes in the large multicenter, randomized control AURORA trial (a study to evaluate the use of rosuvastatin in subjects on regular hemodialysis: an assessment of survival and cardiovascular events). In addition, as PICP and Gal-3 could complementarily investigate ongoing active fibrosis, we investigated their interplay with regard to clinical outcomes, and further attempted to identify a fibrotic biomarker phenotype [23].

## Methods

### Study population

The description, baseline data and main results of the AURORA study have been published previously [24–26]. In short, AURORA is a double-blind, randomized, multicenter study involving 2773 men and women aged 50–80 years, who had been treated with maintenance hemodialysis or hemofiltration for at least 3 months. After providing written informed consent, eligible patients were randomly assigned in a 1:1 ratio to receive either rosuvastatin, 10 mg daily or matching placebo.

There was no significant effect of rosuvastatin on the composite primary endpoint of major adverse cardiovascular event (MACE) (i.e., nonfatal myocardial infarction, nonfatal stroke, or death from CV causes) [HR 0.96 (95% CI 0.84–1.11), *p* = 0.59). Rosuvastatin also exhibited no significant effects on the secondary endpoint of all-cause mortality [HR 0.96 (95% CI 0.86–1.07), *p* = 0.51].

### Outcomes

The pre-specified considered outcomes are CV death, all-cause mortality, and the composite primary endpoint of the AURORA trial (MACE). All the recorded events were reviewed and adjudicated by a clinical endpoint committee to ensure consistency of the event diagnosis. The committee members were unaware of the randomized treatment assignments.

### Biomarkers

PICP [reference range, 69–163 ng/mL] was measured in serum samples by an ELISA (Quidel Corporation, Santa Clara, USA). The dynamic range for this assay is 0.2–80 ng/mL and interassay coefficients of variation were 7%.

Gal-3 was measured in serum by a chemiluminescent microparticle immunoassay (CMIA, Abbott GmbH, Wiesbaden, Germany) on an Abbott ARCHITECT i2000 analyzer. Interassay coefficients of variation were 4.4, 5.2, and 1.6% at low, middle and high concentrations.

PICP was available for all the 2349 patients of the AURORA trial and Gal-3 as available for 2343 patients of the study.

### Statistical analysis

Categorical variables are expressed as frequencies (%) and continuous variables are expressed as means ± standard deviation or median (25th and 75th percentiles), depending on the variable distribution.

Associations of PICP and Gal-3 with CV death, all-cause mortality or MACE were assessed using both continuous and categorized variables (tertiles) using Cox models. Models were adjusted on clinical and biological covariates based on previously published data in AURORA [23] (age, history of CV disease, diabetes mellitus, albumin, and high sensitivity C-reactive protein), and variables correlated to PICP and Gal-3 concentrations (sex, dialysis vintage, body mass index, systolic blood pressure). History of CV disease is defined by history of coronary heart disease (i.e., prior myocardial infarction, prior coronary angioplasty or stent, and coronary artery bypass graft), history of vascular disease (i.e., peripheral artery disease, abdominal aortic aneurysm, carotid artery disease, carotid stenosis ≥ 50%, and carotid endarterectomy), and history of neurovascular disease (i.e., prior ischemic vascular accident and transient ischemic attack). High sensitivity C-reactive protein (hs-CRP) was best modeled by using its natural logarithm. Interaction between PICP and Gal-3 was assessed using a multiplicative interaction term in the Cox models.

The added prognostic value of PICP and Gal-3 in predicting CV death or all-cause mortality was assessed by the net reclassification improvement (NRI) on top of routine prognostic variables used as adjustment variables (i.e., age, history of CV disease, diabetes mellitus, sex, dialysis vintage, body mass index, systolic blood pressure, albumin and log hs-CRP at baseline).

All analyses were performed using R version 3.6.1 (R Development Core Team, Vienna, Austria). The two-sided significance level was set at *p* value < 0.05.

## Results

### Baseline characteristics of patients across the tertiles of each biomarker

The mean PICP was 176 ± 91 ng/mL and mean Gal-3 concentration was 69 ± 25 ng/mL. The correlation between PICP and Gal-3 was weak (Pearson correlation 0.068, *p* = 0.002). The correlation between hs-CRP and Gal-3 was also weak (Pearson correlation 0.124, *p* < 0.0001), whereas there was no correlation between hs-CRP and PICP (Pearson correlation − 0.009, *p* = 0.64).

Patients with higher PICP concentrations were younger (median age 63 in tertile (*T*) 3 vs. 66 in *T*1, *p* < 0.001) and had longer dialysis vintage (median 3.88 years vs. 2.04, *p* < 0.001). Concentrations of hs-CRP were similar across the PICP tertiles. Patients with higher PICP concentrations were more likely to be women (*p* < 0.001) and had lower BMI (*p* < 0.001), and lower hemoglobin levels (*p* < 0.001) (Table 1).

**Table 1:** Baseline characteristics according to PICP tertiles

PICP (ng/mL)	1st tertile (11.3–130)	2nd tertile (> 130–186)	3rd tertile (> 186–800)	*p *value
*n*	**783**	**784**	**782**	
Female gender (%)	271 (34.6)	284 (36.2)	343 (43.9)	** < 0.001**
Age (years)	66 [58–73]	66 [57–73]	63 [56–71]	** < 0.001**
Dialysis vintage (years)	2.04 [0.91–4.35]	2.53 [1.05–4.86]	3.88 [1.77–7.69]	** < 0.001**
Measured (K_t_/V)	1.36 [1.19–1.58]	1.36 [1.20–1.56]	1.36 [1.19–1.59]	0.915
Albumin (g/L)	39.73 (3.35)	39.71 (3.47)	39.37 (3.53)	0.065
Hemoglobin (g/dL)	11.89 (1.51)	11.78 (1.53)	11.45 (1.65)	** < 0.001**
hs-CRP (mg/L)	1.01 (1.14)	1.02 (1.17)	1.01 (1.18)	0.984
BMI (kg/m^2^)	25.53 (4.90)	25.73 (5.05)	24.71 (4.73)	** < 0.001**
Systolic blood pressure (mmHg)	134 (23.15)	135 (24.01)	138 (25.46)	**0.003**
Diastolic blood pressure (mmHg)	74 (12.35)	75 (12.11)	77 (13.01)	** < 0.001**
Pulse pressure (mmHg)	60 [49–70]	60 [46–71]	60 [50–73]	0.368
Current smoker (%)	139 (17.8)	123 (15.7)	116 (14.8)	0.271
Diabetes (%)	199 (25.4)	200 (25.5)	190 (24.3)	0.827
Peripheral artery disease (%)	136 (17.4)	111 (14.2)	116 (14.8)	0.179
History of coronary heart disease (%)	106 (13.5)	106 (13.5)	100 (12.8)	0.883
History of cardiovascular disease (%)	284 (36.3)	270 (34.4)	237 (30.3)	**0.038**
Cause of ESRD, *n* (%)				0.088
Diabetes	136 (17.4)	146 (18.6)	133 (17.0)	
Genetic conditions	100 (12.8)	106 (13.5)	104 (13.3)	
Glomerulonephritis or vasculitis	126 (16.1)	160 (20.4)	173 (22.1)	
Nephropathy or nephrosclerosis	180 (23.0)	145 (18.5)	134 (17.1)	
Pyelonephritis or interstitial	119 (15.2)	109 (13.9)	128 (16.4)	
Unknown/unspecified	80 (10.2)	75 (9.6)	76 (9.7)	
Other	42 (5.4)	43 (5.5)	34 (4.3)	

*hs-CRP* high sensitivity C-reactive protein, *BMI* body mass index, *ESRD* end-stage renal disease

Results with *p* value less than 5% were emphasized using bold letters

Patients with higher Gal-3 concentrations were younger (median age 63 in *T*3 vs. 65 in *T*1, *p* = 0.008), had longer dialysis vintage (median 3.82 years vs. 1.89, *p* < 0.001) and higher levels of hs-CRP (*p* < 0.001) (Table 2).

**Table 2:** Baseline characteristics according to Gal-3 tertiles

Gal-3 (ng/mL)	1st tertile (11.2–56.3)	2nd tertile (> 56.3–78)	3rd tertile (> 78–228)	*p* value
*N*	**782**	**780**	**781**	
Female gender (%)	284 (36.3)	291 (37.3)	313 (40.1)	0.283
Age (years)	65 [57–73]	65 [57–72]	63 [56–71]	**0.008**
Dialysis vintage (years)	1.89 [0.83–4.02]	2.87 [1.24–5.34]	3.82 [1.78–7.01]	** < 0.001**
Measured (K_*t*_/V)	1.34 [1.20–1.56]	1.38 [1.19–1.60]	1.36 [1.20–1.56]	0.508
Albumin (g/L)	39.73 (3.44)	39.95 (3.46)	39.26 (3.53)	** < 0.001**
Hemoglobin (g/dL)	11.76 (1.49)	11.68 (1.63)	11.57 (1.66)	0.067
hs-CRP (mg/L)	0.88 (1.07)	0.95 (1.12)	1.22 (1.27)	** < 0.001**
BMI (kg/m^2^)	25.16 (4.82)	25.40 (4.64)	25.31 (5.30)	0.618
Systolic blood pressure (mmHg)	138 (21.69)	138 (25.01)	136 (26.32)	0.342
Diastolic blood pressure (mmHg)	76 (12.12)	76 (12.36)	76 (13.51)	0.867
Pulse pressure (mmHg)	60 [50–72]	60 [49–74]	60 [48–70]	0.100
Current smoker (%)	128 (16.4)	117 (15.0)	117 (15.0)	0.685
Diabetes (%)	220 (28.1)	197 (25.3)	187 (23.9)	0.153
Peripheral artery disease (%)	113 (14.5)	124 (15.9)	108 (13.8)	0.496
History of coronary heart disease (%)	102 (13.0)	103 (13.2)	93 (11.9)	0.704
History of cardiovascular disease (%)	262 (33.5)	256 (32.8)	241 (30.9)	0.522
Cause of ESRD, *n* (%)				0.119
Diabetes	155 (19.8)	141 (18.1)	136 (17.4)	
Genetic conditions	101 (12.9)	111 (14.2)	89 (11.4)	
Glomerulonephritis or vasculitis	126 (16.1)	133 (17.1)	163 (20.9)	
Nephropathy or nephrosclerosis	161 (20.6)	156 (20.0)	151 (19.3)	
Pyelonephritis or interstitial	131 (16.8)	115 (14.7)	115 (14.7)	
Unknown/unspecified	67 (8.6)	94 (12.1)	91 (11.7)	
Other	41 (5.2)	30 (3.8)	36 (4.6)	

*BMI* body mass index, *ESRD* end-stage renal disease

Results with *p* value less than 5% were emphasized using bold letters

The hs-CRP (mg/L) tertiles are presented in Supplementary Table 1.

### Association of PICP, Gal-3, and hs-CRP with CV mortality, all-cause mortality, and MACE

In multivariable analysis (Table 3), after adjusting for key prognostic factors (including age which was inversely correlated to PICP concentrations), PICP was associated with CV death [HR per 1 SD = 1.11 (1.02–1.20)]. PICP was also significantly associated with all-cause mortality [HR per 1 SD = 1.12 (1.05–1.19) and HR for *T*3 vs. *T*1 = 1.26 (1.08–1.46)] (Table 3).

**Table 3:** Association of PICP, Gal-3 and hs-CRP with cardiovascular death and all-cause mortality

Variables	Univariable HR (95% CI)	*p* value	Multivariable			
			Model 1 HR (95% CI)	*p* value	Model 2 HR (95% CI)	*p* value
Cardiovascular death (*n* = 648)						
PICP						
Per 1SD	1.06 (0.98–1.15)	0.13	1.12 (1.03–1.21)	**0.01**	1.11 (1.02–1.20)	**0.017**
Tertiles						
1st tertile	1	–	1	–	1	–
2nd tertile	1.00 (0.82–1.23)	0.98	1.01 (0.82–1.25)	0.89	1.01 (0.82–1.24)	0.96
3rd tertile	1.14 (0.93–1.39)	0.20	1.22 (0.99–1.50)	0.061	1.22 (0.99–1.50)	0.068
Gal-3						
Per 1SD	1.15 (1.06–1.24)	**0.0006**	1.23 (1.15–1.31)	** < 0.0001**	1.20 (1.10–1.31)	** < 0.0001**
Tertiles						
1st tertile	1	–	1	–	1	–
2nd tertile	1.11 (0.91–1.36)	0.31	1.16 (0.94–1.42)	0.16	1.14 (0.93–1.40)	0.21
3rd tertile	1.29 (1.06–1.57)	**0.012**	1.46 (1.19–1.79)	**0.0003**	1.36 (1.11–1.67)	**0.003**
hs-CRP^a^						
Log hs-CRP	1.24 (1.16–1.32)	** < 0.0001**	1.22 (1.15–1.30)	** < 0.0001**	1.17 (1.10–1.26)	** < 0.0001**
Tertiles						
1st tertile	1	–	1	–	1	–
2nd tertile	1.14 (0.94–1.40)	0.19	1.14 (0.93–1.40)	0.20	1.09 (0.89–1.34)	0.39
3rd tertile	1.66 (1.38–2.01)	** < 0.0001**	1.62 (1.33–1.97)	** < 0.0001**	1.42 (1.16–1.73)	**0.0008**
All-cause mortality (*n* = 1296)						
PICP						
Per 1SD	1.07 (1.01–1.13)	**0.021**	1.12 (1.06–1.19)	**0.0002**	1.12 (1.05–1.19)	**0.0002**
Tertiles						
1st tertile	1	–	1	–	1	–
2nd tertile	1.09 (0.94–1.26)	0.24	1.09 (0.94–1.26)	0.25	1.09 (0.94–1.26)	0.28
3rd tertile	1.18 (1.02–1.36)	**0.027**	1.26 (1.08–1.46)	**0.003**	1.26 (1.08–1.46)	**0.003**
Gal-3						
Per 1SD	1.14 (1.08–1.21)	** < 0.0001**	1.21 (1.14–1.29)	** < 0.0001**	1.18 (1.10–1.25)	** < 0.0001**
Tertiles						
1st tertile	1	–	1	–	1	–
2nd tertile	1.05 (0.91–1.22)	0.50	1.10 (0.95–1.28)	0.22	1.08 (0.93–1.26)	0.30
3rd tertile	1.23 (1.06–1.42)	**0.006**	1.38 (1.19–1.61)	** < 0.0001**	1.26 (1.08–1.46)	**0.003**
hs-CRP^a^						
Log hs-CRP	1.28 (1.22–1.33)	** < 0.0001**	1.28 (1.22–1.34)	** < 0.0001**	1.22 (1.17–1.28)	** < 0.0001**
Tertiles						
1st tertile	1	–	1	–	1	–
2nd tertile	1.31 (1.14–1.51)	**0.0002**	1.34 (1.16–1.56)	** < 0.0001**	1.29 (1.11–1.49)	**0.0007**
3rd tertile	1.90 (1.66–2.18)	** < 0.0001**	1.91 (1.66–2.20)	** < 0.0001**	1.68 (1.45–1.94)	** < 0.0001**

Model 1: adjusted for age, diabetes, history of cardiovascular disease, sex, dialysis vintage, body mass index, and systolic blood pressure (at baseline)

Model 2: model 1 + albumin and log hs-CRP (at baseline)

^a^Model 2: model 1 + albumin (at baseline)

Results with *p* value less than 5% were emphasized using bold letters

The association of Gal-3 either considered as a continuous variable or categorized variable with CV death and all-cause mortality were significant in multivariable models [adjusted HR per 1 SD = 1.20 (1.10–1.31) and 1.18 (1.10–1.25), respectively; adjusted HR for *T*3 vs. *T*1 = 1.36 (1.11–1.67) and 1.26 (1.08–1.46), respectively] (Table 3).

Gal-3 as a continuous variable was significantly associated with MACE [crude HR per 1 SD = 1.11 (1.03–1.19) and adjusted HR per 1SD = 1.15 (1.07–1.24)]. However, PICP either considered as a continuous variable or categorized variable was not significantly associated with MACE (Supplementary Table 2). Age, diabetes, history of CV disease, as well as a low serum albumin or an elevated hs-CRP were also significantly associated with MACE in the models (Supplementary Table 3).

When further including an interaction term between biomarkers tertiles (either PICP or Gal-3) and dialysis vintage in the multivariable models, we did not identify a significant modification of the associations of biomarkers with CV death, all-cause mortality and MACE related to dialysis vintage (*p* for interaction > 0.05, data not shown). Also, further including the underlying cause of ESRD in the multivariable models did not significantly change the association of biomarkers (either PICP or Gal-3) as a continuous variable or categorized variable with CV death, all-cause mortality, and MACE (data not shown).

**Fig. 1:**
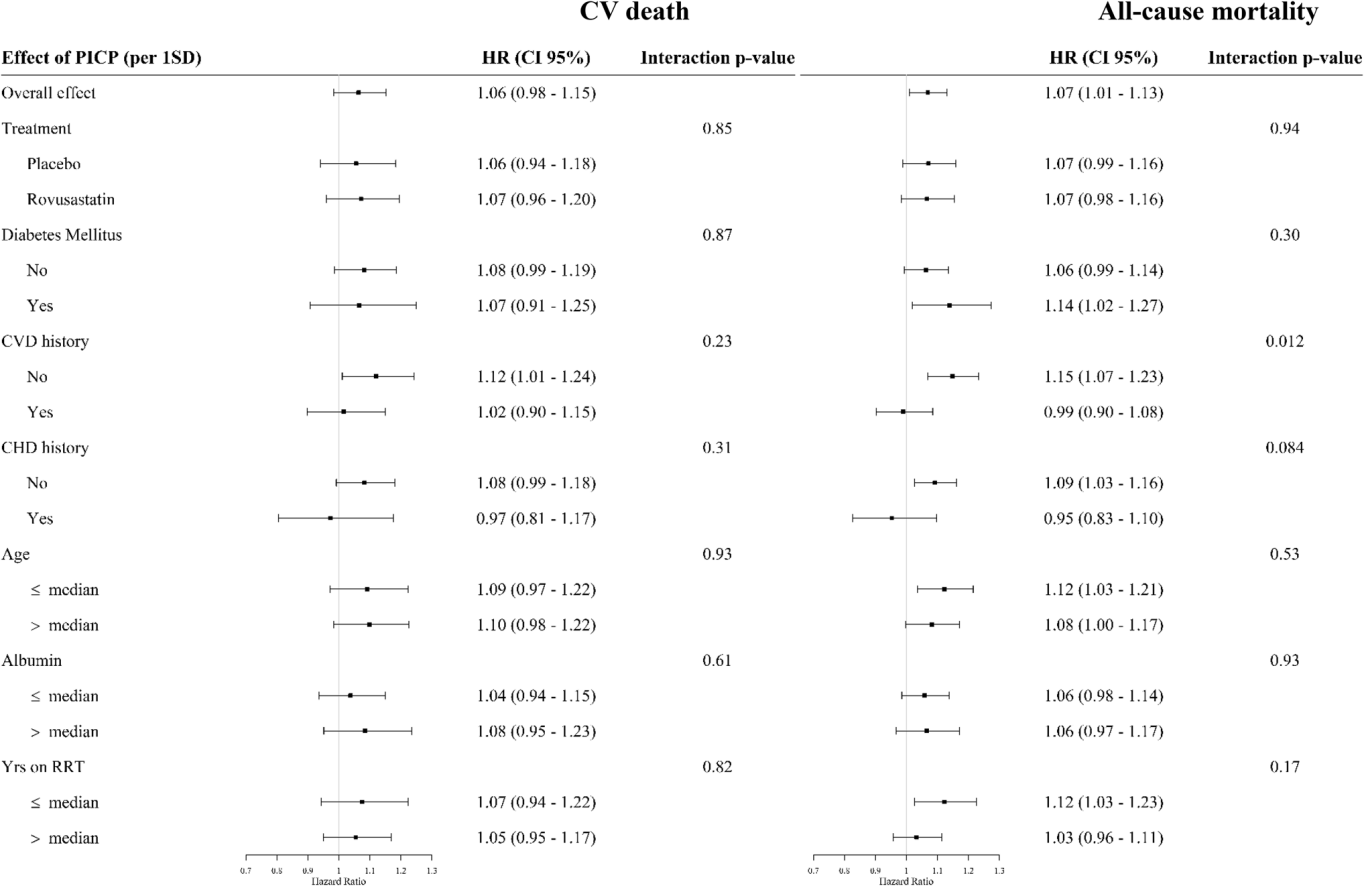
Association between PICP and cardiovascular death or all-cause mortality in subgroups of patients (non-adjusted analysis). *CVD* cardiovascular diseases, *CHD* coronary heart diseases, *Yrs RRT* years on renal replacement therapy (dialysis vintage)

**Fig. 2:**
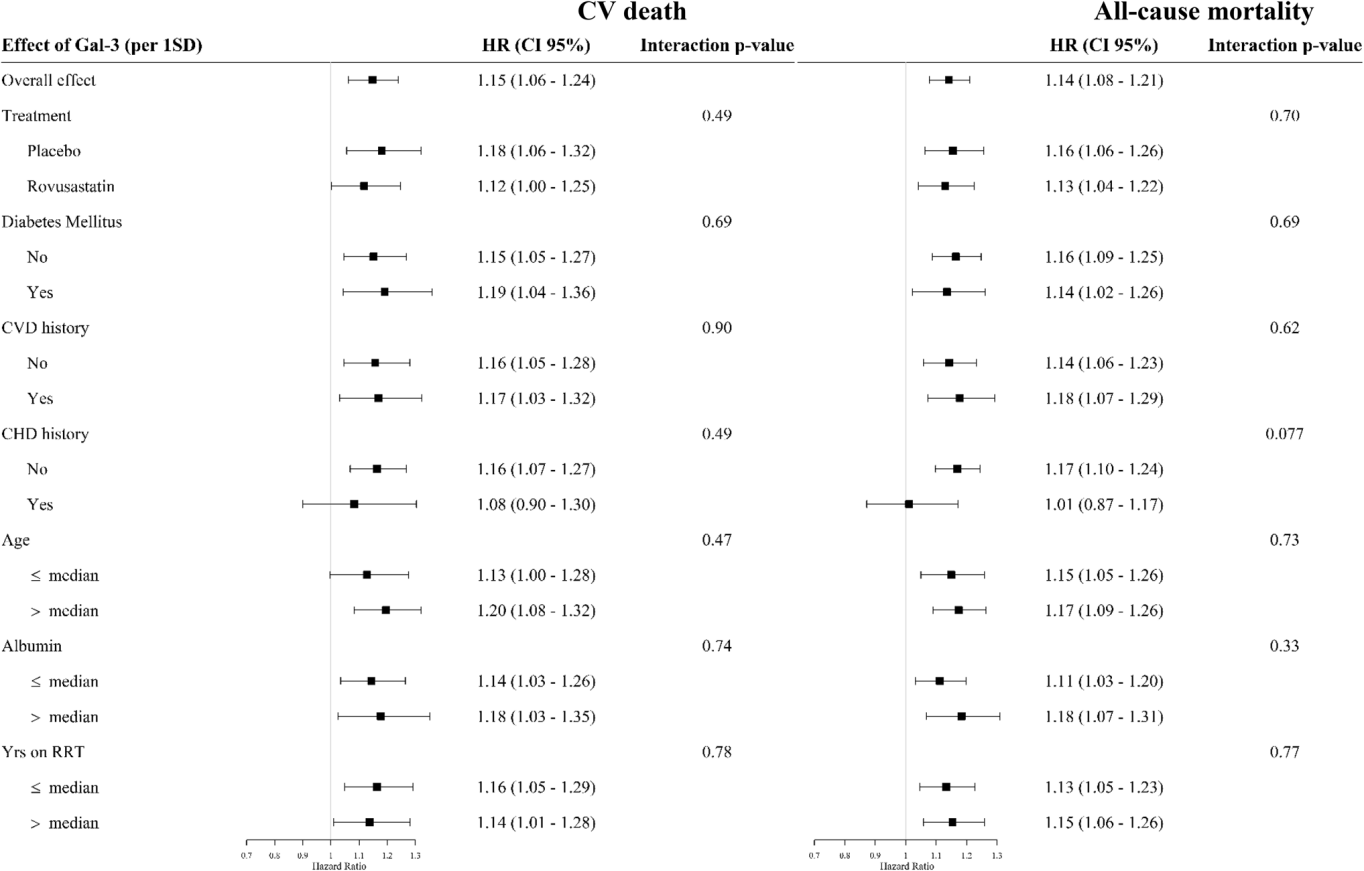
Association between Gal-3 and cardiovascular death or all-cause mortality in subgroups of patients (non-adjusted analysis). *CVD* cardiovascular diseases, *CHD* coronary heart diseases, *Yrs RRT* years on renal replacement therapy (dialysis vintage)

The effect of PICP (per 1-SD) and Gal-3 (per 1-SD) on CV death was homogenous across pre-specified subgroups. In contrast, the association of PICP with all-cause mortality was stronger in patients without history of CV disease [*p* for interaction = 0.012 (Fig. 1); *p* for interaction in multivariable adjusted analysis = 0.010 (Supplementary Fig. 1)] and without history of coronary diseases [*p* for interaction = 0.084 (Fig. 1); *p* for interaction in multivariable adjusted analysis = 0.048 (Supplementary Fig. 1)]. The association of Gal-3 with all-cause mortality was also stronger in patients without history of coronary diseases [(*p* for interaction = 0.077 (Fig. 2); *p* for interaction in multivariable adjusted analysis = 0.085 (Supplementary Fig. 2).

### Interaction of PICP, Gal-3 and hs-CRP to predict CV mortality, all-cause mortality, and MACE

We identified a significant positive interaction between PICP and Gal-3 with regard to CV death and all-cause mortality (*p* for interaction with continuous variables = 0.04 and 0.01, respectively).

**Fig. 3:**
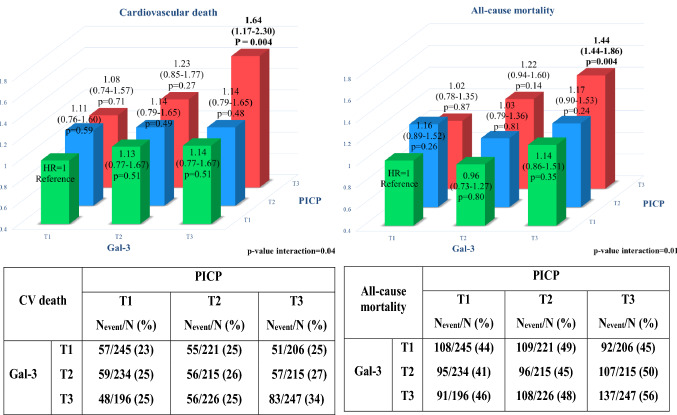
Interaction between PICP and Gal-3 (in tertiles) for the association with CV death and all-cause mortality

The magnitude of the association of an elevated concentration of PICP with either CV death or all-cause mortality was more pronounced among patients having also an elevated concentration of Gal-3 (Fig. 3). Patients with both raised PICP and Gal-3 had sizeable increase in rates for CV death (HR in patients in *T*3 of PICP and *T*3 of Gal-3 = 1.64, *p* = 0.004), whereas patients with isolated Gal-3 or PICP increase, respectively, had no significant increase in rates of CV death (all *p* > 0.20, Fig. 3). In contrast, there was no significant interaction between hs-CRP and PICP regarding the association with either CV death or all-cause mortality (Supplementary Fig. 3).

Interaction between PICP and Gal-3 with regard to MACE tended to be significant (*p* = 0.055), and only patients with elevated Gal-3 and PICP had significantly increased from the risk of MACE [HR = 1.36 (1.00–1.84), Supplementary Fig. 4].

There was no significant interaction between rosuvastatin therapy and PICP and Gal-3 concentration (data not shown).

### Added value of PICP, Gal-3 and hs-CRP to predict CV mortality and all-cause mortality

PICP per 1 SD increase significantly improved the prediction [NRI = 0.072 (0.012–0.115)] of all-cause mortality on top of the routine clinical and biological model based on the variables used for adjustment (i.e., age, history of CV disease, diabetes mellitus, sex, dialysis vintage, body mass index, systolic blood pressure, albumin and log hs-CRP at baseline). Gal-3 significantly improved the prediction of both CV death and all-cause mortality on top of the clinical and biological model [NRI = 0.101 (0.024–0.146) and NRI = 0.080 (0.015–0.113), respectively].

Adding PICP, Gal-3 and their interaction on top of the clinical and biological model (i.e., age, history of CV disease, diabetes mellitus, sex, dialysis vintage, body mass index, systolic blood pressure, albumin and log hs-CRP at baseline) resulted in significant NRI for CV death and all-cause mortality [NRI = 0.080 (0.019–0.143) and 0.085 (0.029–0.129), respectively]. Of note, this prediction improvement was of similar magnitude to the one derived from hs-CRP on top of usual clinical/biological variables (i.e., age, history of CV disease, diabetes mellitus, sex, dialysis vintage, body mass index, systolic blood pressure, and albumin but obviously excluding log hs-CRP at baseline) [NRI for CV death = 0.108 (0.050–0.167) and NRI for all-cause mortality = 0.132 (0.088–0.171), respectively].

## Discussion

To the best of our knowledge, the current study is the first to assess the combined and synergistic association of PICP and Gal-3 with CV death and all-cause mortality in hemodialysis patients. Our main findings are as follows: (1) increased concentrations of PICP or Gal-3 are significantly associated with CV death and all-cause mortality even after adjusting on a previously validated clinical and biological score in the AURORA trial [23], and the correlated variables; (2) the increment of association with CV death provided by PICP and Gal-3 was of similar magnitude of the one provided by hs-CRP [HR per 1 SD 1.11 (1.02–1.20) = 1.20 (1.10–1.31) and 1.17 (1.10–1.26) for PICP, Gal-3, and hs-CRP; respectively]; (3) there is a positive interaction between Gal-3 and PICP regarding the association with CV death and all-cause mortality whereas no significant interaction is found between PICP and hs-CRP.

The pathophysiology of CV complications is very complex in patients on dialysis, but chronic inflammation certainly plays a major role [3, 27, 28]. Moreover, cardiac and vascular fibrosis are likely to be the main histological pathways involved in the generation of CV complications related to uremic cardiomyopathy and vascular stiffness [7].

Uremic cardiomyopathy refers to histological modifications of the heart, generated by chronic fluid overload, chronic mineral bone disorder, as well of chronic inflammation, all of them ultimately leading to cardiac fibrosis [4]. Arterial stiffness is associated with an increased mortality in ESRD [29], and it is now well-established that fibrosis also is the main histological finding of this vascular pathology [6].

Outside the field of CKD, PICP has been shown to be raised in HF, hypertensive or ischemic heart disease with or without HF, and arterial stiffness [9, 14]. Moreover, in HF, serum concentrations of PICP are associated with worse prognosis [30–32], but little is known concerning its clinical risk prediction in dialysis.

The catabolism of high molecular weight PICP is mainly hepatic [33]. Thus, PICP does not accumulate as a consequence of impaired kidney function. Moreover, among prevalent dialysis patients, the concentration of PICP unlikely reflects active renal fibrosis, as patients have established ESRD for long periods. Finally, patients with liver cirrhosis were excluded from the AURORA trial [24]. Consequently, we hypothesized in this study that the concentration of PICP may partly reflect cardiac and vascular fibrosis. Indeed, increased PICP concentrations indicate diastolic dysfunction in ESRD patients undergoing chronic dialysis [34].

Gal-3, a 29–35 kDa protein, is a member of the β-galectin binding lectin family, which is mainly secreted by macrophages, fibroblasts, mast cells and neutrophils [35]. Gal-3 plays a major role in the pathophysiology of HF, as a marker and a mechanism of inflammation potentially leading to fibrosis [36], but little is known about the implication of Gal-3 in the CV complications among patients on dialysis. Gal-3 appears to be related to chronic systemic inflammation [22, 37].

Of note, the interplay between Gal-3 and collagen biomarkers has not been evaluated in the setting of dialysis. The association of Gal-3 and CV outcome in patients with chronic kidney disease (CKD) and ESRD, was previously reported in a pooled analysis of the LURIC (Ludwigshafen risk and cardiovascular health) and the 4D (die deutsche diabetes dialyse studie) trial. The authors reported that elevated Gal-3 was significantly associated with CV death and all-cause mortality among patients with CKD and ESRD [19]. Gal-3 plays a pivotal role in the inflammatory response by binding to the extracellular matrix (ECM) proteins and modulating adhesions of the immune cells, including T cells, neutrophils, monocytes, and mast cells [38, 39]. In preclinical models, the overexpression of Gal-3 in rats enhanced collagen type I synthesis leading to aldosterone-induced vascular inflammation, remodeling, and fibrosis [40]. Vergaro et al. supported the role of Gal-3 in the development of myocardial inflammation and fibrogenesis. In their study, they showed that inhibition of Gal-3 in mice could reverse drug-induced left ventricular dysfunction by reducing myocardial inflammation and fibrosis [41]. Martinez-Martinez et al. reported in a preclinical study that Gal-3 expression is upregulated by cardiotrophin-1 (CT-1) which mediates the proinflammatory and profibrotic myocardial effects [35, 40, 42–45].

In the general population, the mean Gal-3 concentrations fluctuate between 11 and 14 ng/mL [16, 46–48], whereas they have been reported to be as high as 54 ng/mL in the 4D patients undergoing hemodialysis [19]. Similar to the latter study, the mean Gal-3 concentration in our study was 69 ng/mL. Moreover, Gal-3 concentrations reach up to 26 ng/mL in congestive heart failure (CHF) [49–51]. This association between Gal-3 and kidney function may explain the increased oxidative stress partially explaining chronic inflammation. PICP, Gal-3, and hs-CRP concentrations in the current dialysis population were compared to other categories of high CV risk patients [19, 48, 52–55], as presented in Table 4.

**Table 4:** CRP, PICP, and Gal-3 concentrations in the AURORA population in comparison to other populations of high CV risk patients

Reference	Studied conditions	CRP (mg/L)	PICP (ng/mL)	Gal-3 (ng/mL)
Eschalier et al*.* [52] Tarjus et al*.* [48]	Abdominal obesity	4.0 ± 6.2	87.0 ± 52.0	12.2 (10.9–15.0)
Barasch et al*.* [53] Gopal et al*.* [54]	HFrEF	3.3 (1.5–7.7)	406 (353–477)	23.0 ± 12.0
Barasch et al*.* [53] Gopal et al*.* [54]	HFpEF	4.1 (1.4–9.1)	395 (329–503)	22.0 ± 10.0
Drechsler et al*.* [19]	CKD	6.5 (2.5–14.9)	N/A	23.1 ± 9.9
Drechsler et al*.* [19] Sawamura et al*.* [55]	Hemodialysis	10.6 ± 17.2	162.0 ± 66.0	54.1 ± 19.6
Current study (AURORA)	Hemodialysis	1.0048 ± 1.16 (hs-CRP)	175.9 ± 91.2	69.3 ± 25.1

*HFrEF* heart failure with reduced ejection fraction, *HFpEF* heart failure with preserved ejection fraction, *CKD* chronic kidney disease, *N/A* not applicable

Excessive collagen production, fibroblasts and accumulation of ECM are stimulated by the high concentrations of Gal-3 within the myocardium [14, 21, 56]. This explains the significant interaction between PICP and Gal-3 with regard to CV death and all-cause mortality. The synergistic association of PICP and Gal-3 on mortality, in AURORA study, suggests that in dialysis patients, the process of active fibrosis was driven by Gal-3 overexpression and oxidative stress, more specifically than by chronic inflammation [57]. Indeed, the association between PICP and mortality was found only in dialysis patients without history of CV disease and coronary heart disease, and, importantly, no interaction was found between PICP and hs-CRP.

Importantly, PICP was more associated with CV death than morbid events, as there was no significant association between PICP and MACE. In addition, Gal-3 was less associated with MACE than with CV death, further strengthening the relevance of these biomarkers to predict mortality rather than morbidity.

Patients with ESRD suffer from uncontrolled secondary hyperparathyroidism where PICP concentrations may reflect bone degradation [58]. Although this mechanism may account for some of the rise in PICP in our dialysis patients, it is unlikely to be prominent since it would not explain the significant interaction found between Gal-3 and PICP. This interaction may reflect an active process of fibrosis, which is probably more often observed among younger patients in dialysis, without history of coronary heart disease.

### Clinical implications

The combined use of PICP and Gal-3, as a non-invasive assessment of fibrosis, could improve CV risk prediction on top of validated clinical and biological risk scores in patients undergoing chronic hemodialysis. Hence, the identification of patients with active fibrosis may be a promising approach as they probably have the highest probability to benefit from anti-fibrotic interventions, similarly to what is performed among non-dialyzed patient at risk of heart failure [59]. Yet, the application of this strategy will depend on the prospective validation of a panel of circulating markers in a large-scale population, and the stratification of the latter according to their fibrosis’ profiles and response to a personalized therapy.

In addition, we tested the hypothesis of possible interaction between rosuvastatin therapy on PICP and Gal-3 concentrations [60, 61], and we did not identify any significant interaction in this post hoc setting. However, this does not necessarily mean that other therapeutic intervention directly targeting fibrosis could not be proposed, based on these biomarkers in the future.

Of note, the anti-fibrotic drug class prototype of the mineralocorticoid receptors antagonists is currently tested in two multicenter randomized study CV prevention studies in hemodialysis achieve (aldosterone blockade for health improvement evaluation in end-stage renal disease trial; NCT03020303), and alchemist (aldosterone antagonist chronic hemodialysis interventional survival trial; NCT01848639) [62].

Our work suggests that Gal-3 may be a key mechanistic pathway underlying fibrosis in dialysis patients, anti-Gal3 therapy for CV prevention, such as with modified citrus pectin (MCP) may be worth investigating in this setting. As a competitive inhibitor of Gal-3, MCP binds to intracellular and extracellular Gal-3, reduces level of Gal-3 and prevents cardiac fibrosis, inflammation and functional alterations associated in a number of experimental animal models [35, 40, 43].

## Limitations

As in most blood biomarkers studies, it is not possible to firmly validate the fact that the amount of circulating PICP only reflects cardiac and vascular fibrosis. The measurement of parathyroid hormone (PTH) was not carried out among the patients of AURORA study. It would have been insightful in excluding the possibility that high concentrations of PICP were not reflecting bone turnover. We did not adjust our analysis on NT-proBNP, which is an important prognostic factor [63]. In this multicenter prospective randomized trial with more than 2000 patients and adjudicated outcomes, it was not possible to assess other potentially relevant fibrosis biomarkers [8, 64]. Finally, dedicated trials are still needed to test as well as to validate more powerful treatment strategies based on PICP and Gal-3 concentrations.

## Conclusion

Increased concentrations PICP and Gal-3 were synergistically associated with both CV death and all-cause mortality in patients undergoing chronic hemodialysis. Their significant synergistic interaction with regard to CV death and all-cause mortality may reflect Gal-3 driven active CV fibrosis. The measurement of these biomarkers could be useful in stratifying patients in dialysis according to their CV risk. The use of these biomarkers to target patients with high probability to benefit from anti-fibrotic, anti-Gal-3 therapy should be further studied.

## Supplementary Information

Below is the link to the electronic supplementary material.

Supplementary file1 (DOCX 3014 KB)
