# Supplementary material for: Serum markers of fibrosis, cardiovascular and all-cause mortality in hemodialysis patients: the AURORA trial
Source: Clin Res Cardiol. 2021 Jun 25;111(6):614–26. doi: 10.1007/s00392-021-01898-9 (PMC9151553; doi:10.1007/s00392-021-01898-9)

**Supplementary Material**

**Supplementary Table 1 Tertiles for hs-CRP (mg/L)**

| **1^st^ tertile (T1)** | **2^nd^ tertile (T2)** | **3^rd^ tertile (T3)** |
| --- | --- | --- |
| 0.0252-0.29 | >0.29-0.843 | >0.843-4 |
| 916 | 913 | 915 |

**Supplementary Table 2 Association between PICP, Gal-3, and hs-CRP with the composite primary endpoint of major adverse CV event (MACE)**

| Variables | Univariable  HR (95% CI) | p-value | Multivariable | | | |
| --- | --- | --- | --- | --- | --- | --- |
|  |  |  | Model 1  HR (95% CI) | p-value | Model 2  HR (95% CI) | p-value |
| **1^◦^ endpoint of CV event (n=804)** | | | | | | |
| **PICP** | | | | | | |
| Per 1SD | 1.02 (0.95 - 1.10) | 0.52 | 1.07 (0.99 - 1.15) | 0.10 | 1.06 (0.98 - 1.15) | 0.15 |
| Tertiles |  |  |  |  |  |  |
| 1^st^ tertile | 1 | - | 1 | - | 1 | - |
| 2^nd^ tertile | 1.08 (0.90 - 1.29) | 0.42 | 1.09 (0.91 - 1.31) | 0.36 | 1.08 (0.90 - 1.29) | 0.42 |
| 3^rd^ tertile | 1.07 (0.89 - 1.28) | 0.48 | 1.12 (0.93 - 1.36) | 0.23 | 1.11 (0.92 - 1.35) | 0.27 |
| **Gal-3** | | | | | | |
| Per 1SD | 1.11 (1.03 - 1.19) | **0.004** | 1.17 (1.09 - 1.26) | **<0.0001** | 1.15 (1.07 - 1.24) | **0.0003** |
| Tertiles |  |  |  |  |  |  |
| 1^st^ tertile | 1 | - | 1 | - | 1 | - |
| 2^nd^ tertile | 1.05 (0.88 - 1.26) | 0.57 | 1.09 (0.91 - 1.31) | 0.37 | 1.06 (0.88 - 1.28) | 0.52 |
| 3^rd^ tertile | 1.18 (0.99 - 1.41) | 0.069 | 1.31 (1.09 - 1.57) | **0.009** | 1.22 (1.02 - 1.47) | **0.034** |
| **hs-CRP*** | | | | | | |
| Log hs-CRP | 1.21 (1.14 - 1.28) | **<0.001** | 1.20 (1.13 - 1.27) | **<0.0001** | 1.15 (1.08 - 1.22) | **<0.0001** |
| Tertiles |  |  |  |  |  |  |
| 1^st^ tertile | 1 | - | 1 | - | 1 | - |
| 2^nd^ tertile | 1.18 (0.98 - 1.40) | 0.074 | 1.16 (0.96 - 1.37) | 0.11 | 1.12 (0.93 - 1.34) | 0.23 |
| 3^rd^ tertile | 1.58 (1.33 - 1.87) | **<0.001** | 1.51 (1.27 - 1.81) | **<0.0001** | 1.35 (1.12 - 1.61) | **0.001** |

**Model 1:** Adjusted for age, diabetes, history of cardiovascular disease, sex, dialysis vintage, body mass index, and systolic blood pressure (at baseline)

**Model 2:** model 1 + albumin and log hs-CRP (at baseline)

***Model 2:** model 1 + albumin (at baseline)

**Supplementary Table 3 Complete PICP and Gal-3 models with the composite primary endpoint of major adverse CV event (MACE) as outcome**

|  | Multivariable | | | |
| --- | --- | --- | --- | --- |
|  | **PICP Model**  HR (95% CI) | p-value | **Gal-3 Model**  HR (95% CI) | p-value |
| **1^◦^ endpoint of CV event (n=804)** | | | | |
| Per 1SD | 1.06 (0.98 - 1.15) | 0.15 | 1.15 (1.07 - 1.24) | **0.0003** |
| **Adjustment Variables** |  |  |  |  |
| Age (years) | 1.03 (1.02 - 1.04) | **<0.0001** | 1.03 (1.02 - 1.04) | **<0.0001** |
| Diabetes Mellitus (1=Yes, 0=No) | 1.49 (1.26 - 1.77) | **<0.0001** | 1.52 (1.29 - 1.80) | **<0.0001** |
| History of cardiovascular disease (1=Yes, 0=No) | 1.41 (1.20 - 1.65) | **<0.0001** | 1.48 (1.27 - 1.74) | **<0.0001** |
| Sex (1=Male, 2=Female) | 0.87 (0.74 - 1.03) | 0.099 | 0.91 (0.78 - 1.07) | 0.25 |
| Dialysis Vintage (years) | 1.01 (0.99 - 1.02) | 0.31 | 1.00 (0.99 - 1.02) | 0.79 |
| Body mass index (kg/m^2^) | 1.00 (0.98 - 1.02) | 0.97 | 0.99 (0.98 - 1.01) | 0.34 |
| Systolic blood pressure (mmHg) | 1.00 (1.00 - 1.01) | 0.20 | 1.00 (1.00 - 1.01) | 0.19 |
| Albumin (g/L) | 0.95 (0.92 - 0.97) | **<0.0001** | 0.95 (0.93 - 0.97) | **<0.0001** |
| log hs-CRP (mg/L) | 1.16 (1.09 - 1.23) | **<0.0001** | 1.15 (1.08 - 1.23) | **<0.0001** |

**Supplementary Fig. 1 Association between PICP and cardiovascular death or all-cause mortality in subgroups of patients (adjusted analysis)**


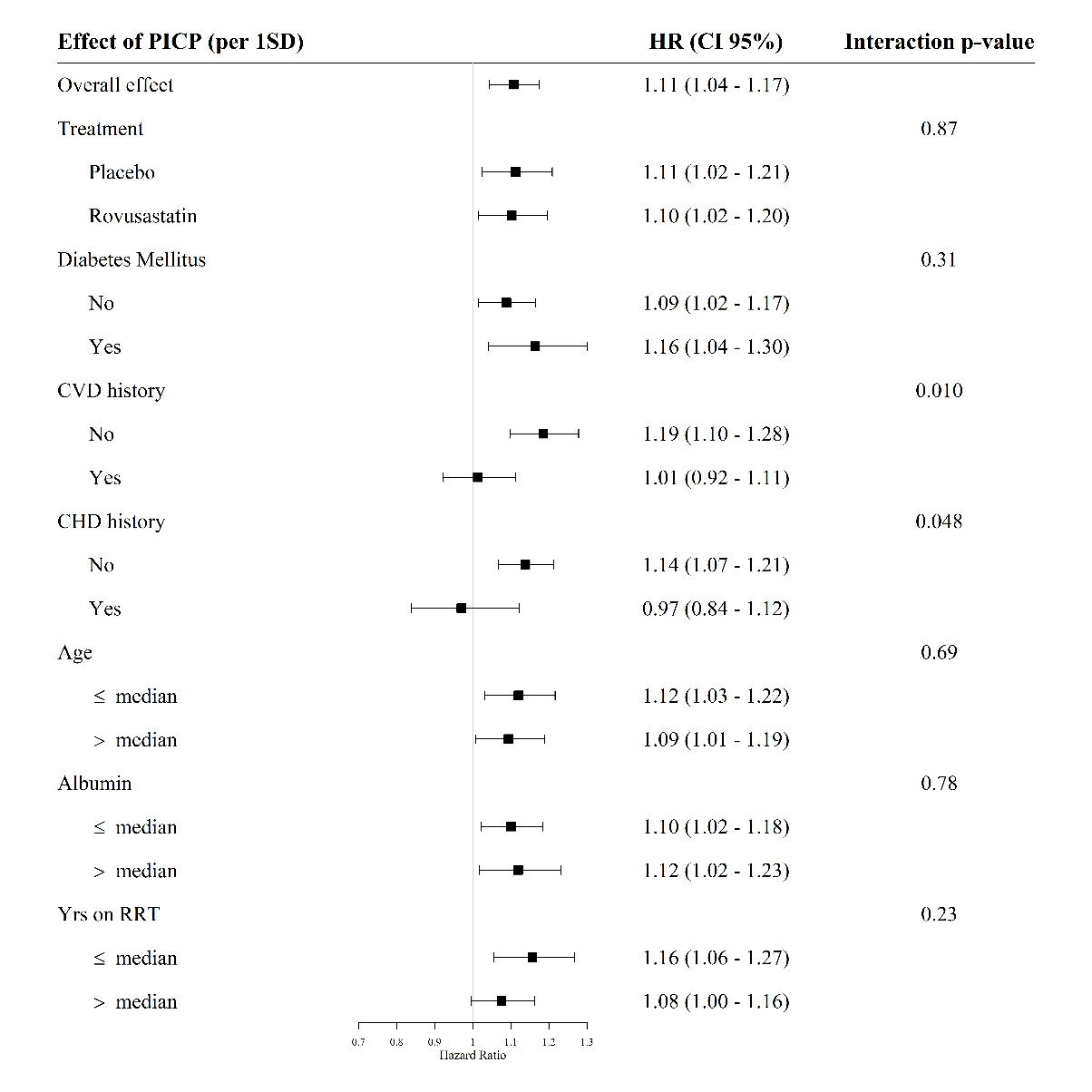

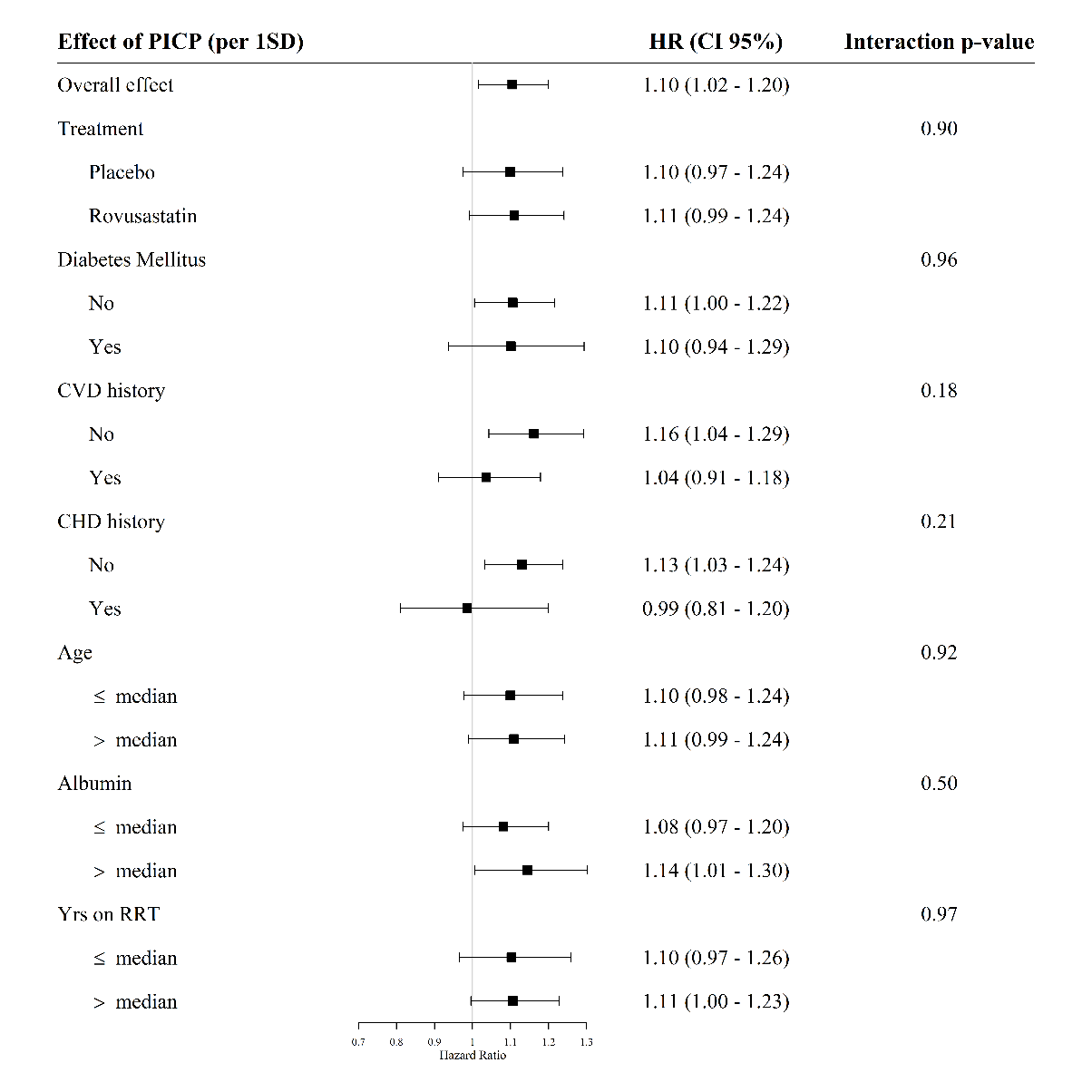


**CV death**

**All-cause mortality**

*CVD, Cardiovascular diseases; CHD, Coronary heart diseases; Yrs on RRT, years on renal replacement therapy (dialysis vintage)*

Adjusted for age, diabetes, history of cardiovascular disease, sex, dialysis vintage, body mass index, systolic blood pressure, albumin, and log

hs-CRP (at baseline).

**Supplementary Fig. 2 Association between Gal-3 and cardiovascular death or all-cause mortality in subgroups of patients (adjusted analysis)**


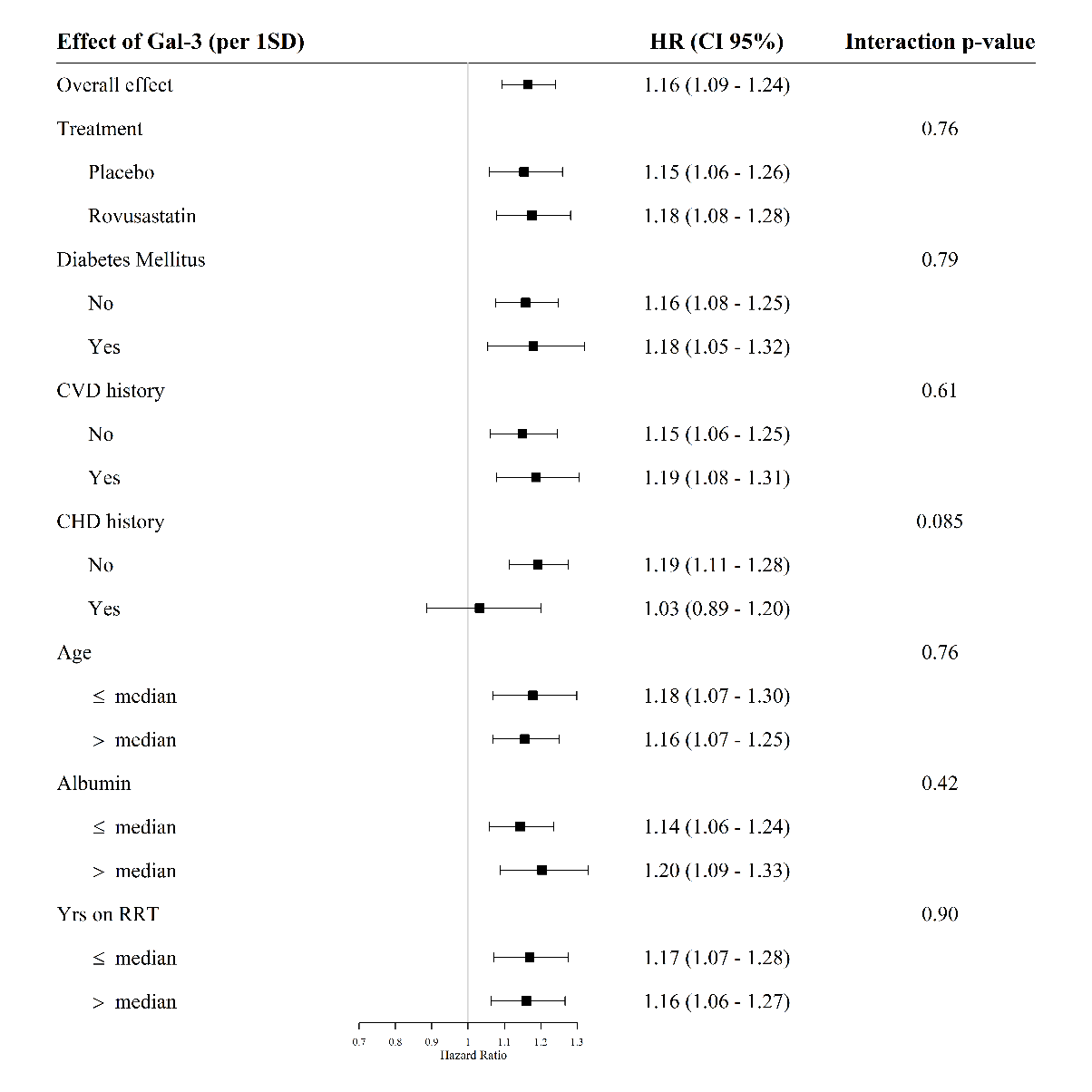

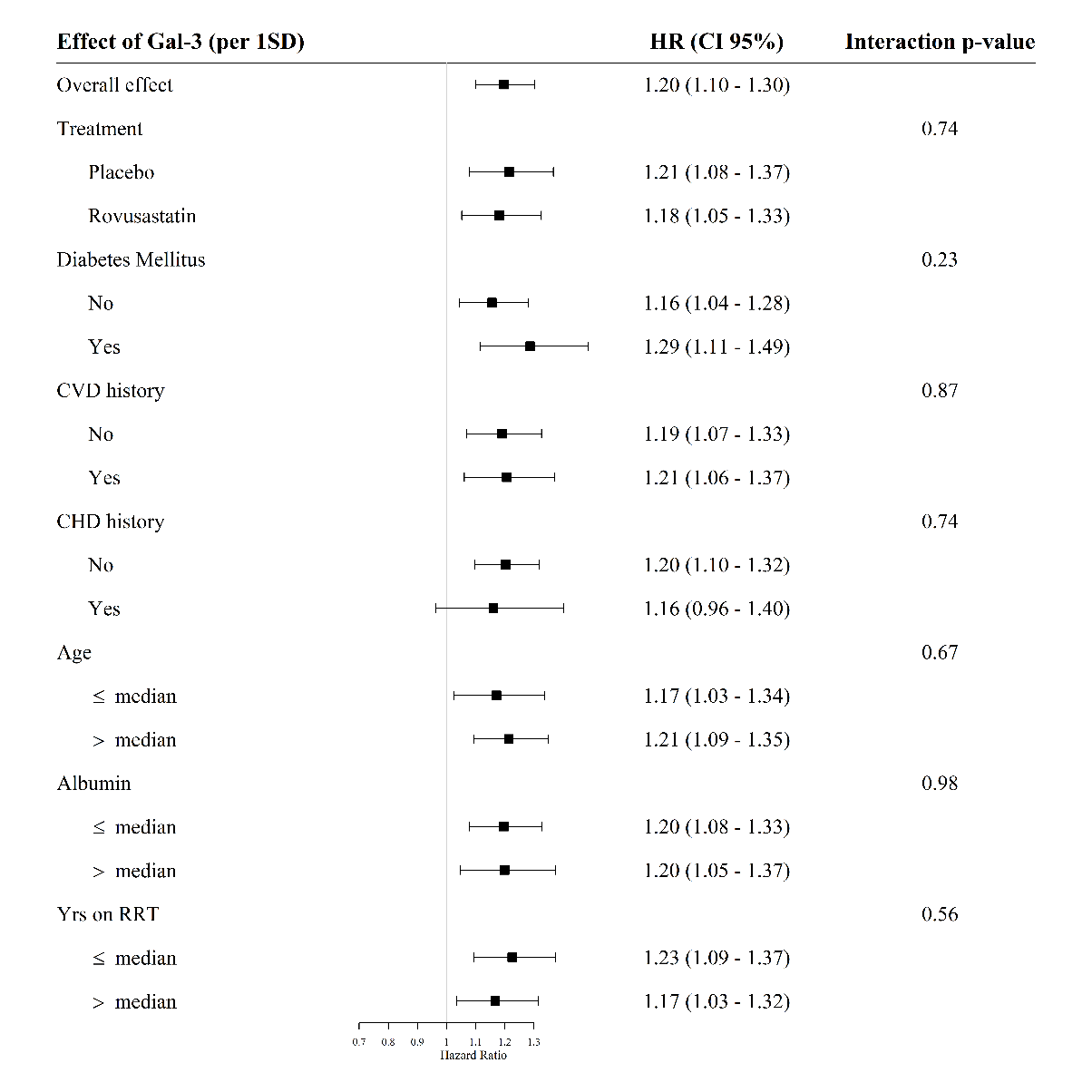


**CV death**

**All-cause mortality**

*CVD, Cardiovascular diseases; CHD, Coronary heart diseases; Yrs on RRT, years on renal replacement therapy (dialysis vintage)*

Adjusted for age, diabetes mellitus, history of cardiovascular disease, sex, dialysis vintage, body mass index, systolic blood pressure, albumin, and log hs-CRP (at baseline).

**Supplementary Fig. 3 Interaction between PICP and hs-CRP (in tertiles) for the association**

**with CV death and all-cause mortality**


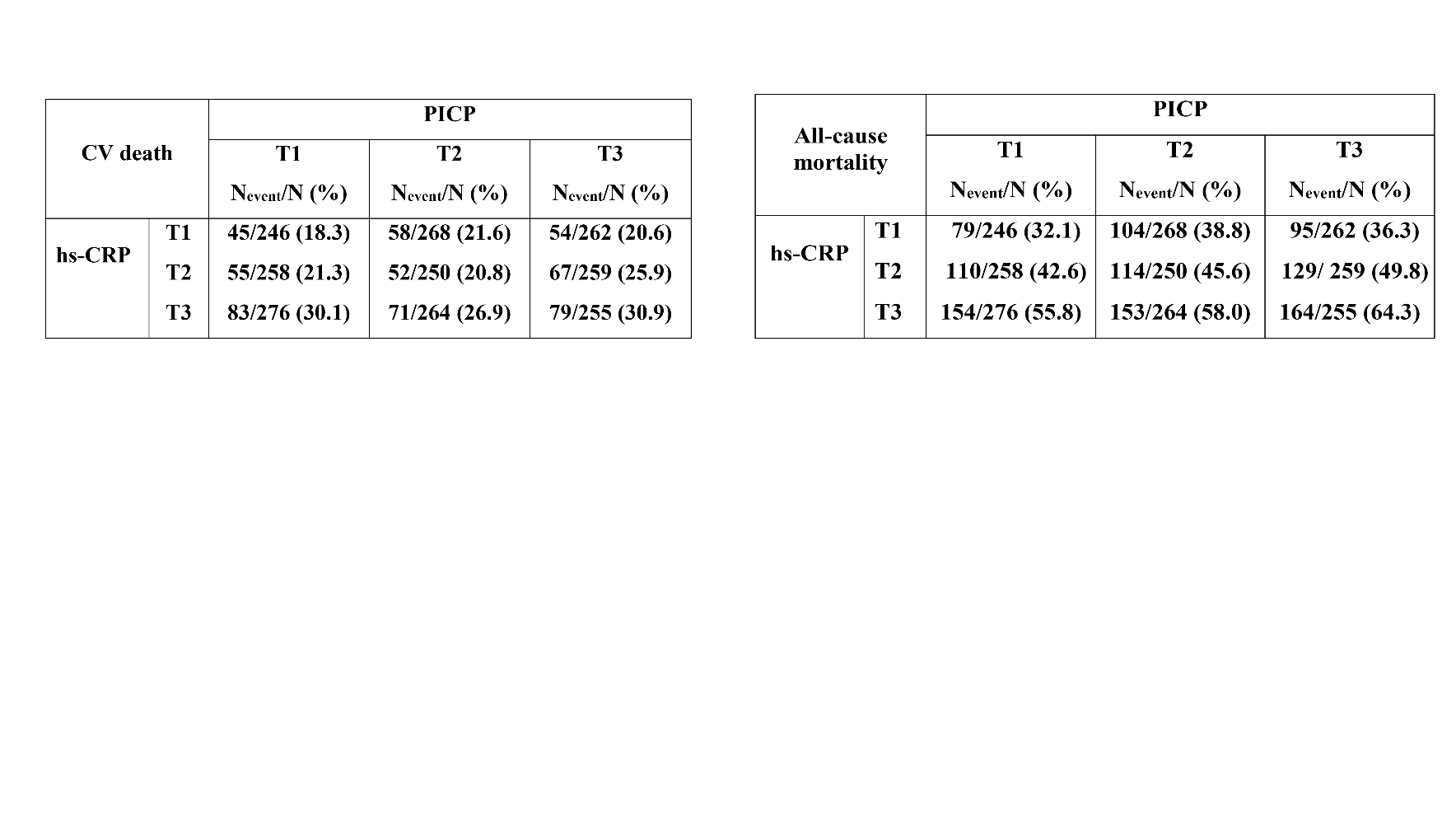

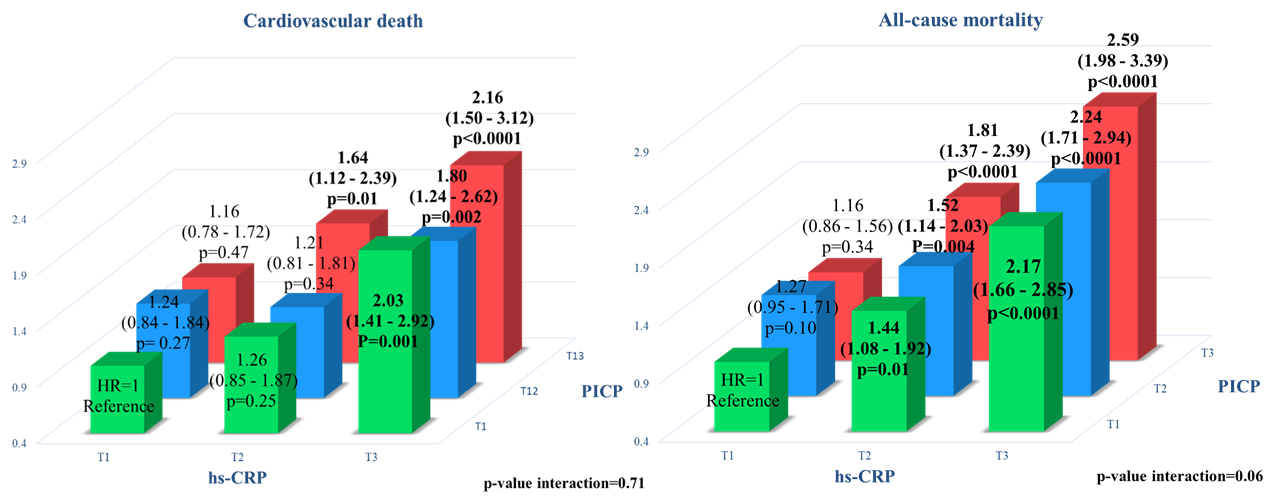


**Supplementary Fig. 4 Interaction between PICP and Gal-3 (in tertiles) for the association with the composite primary endpoint of major adverse CV event (MACE) (non-adjusted analysis)**


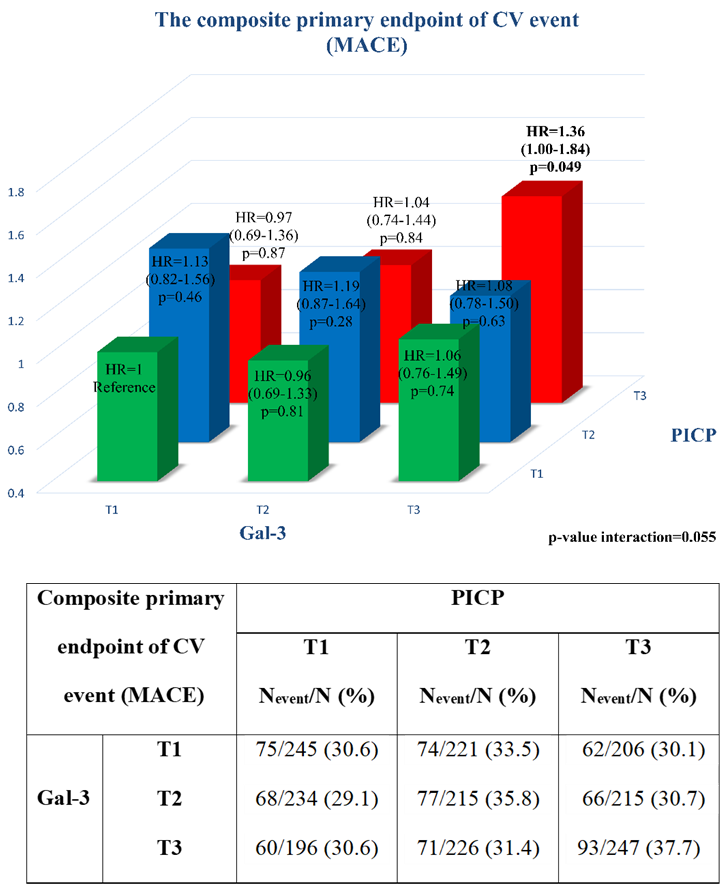

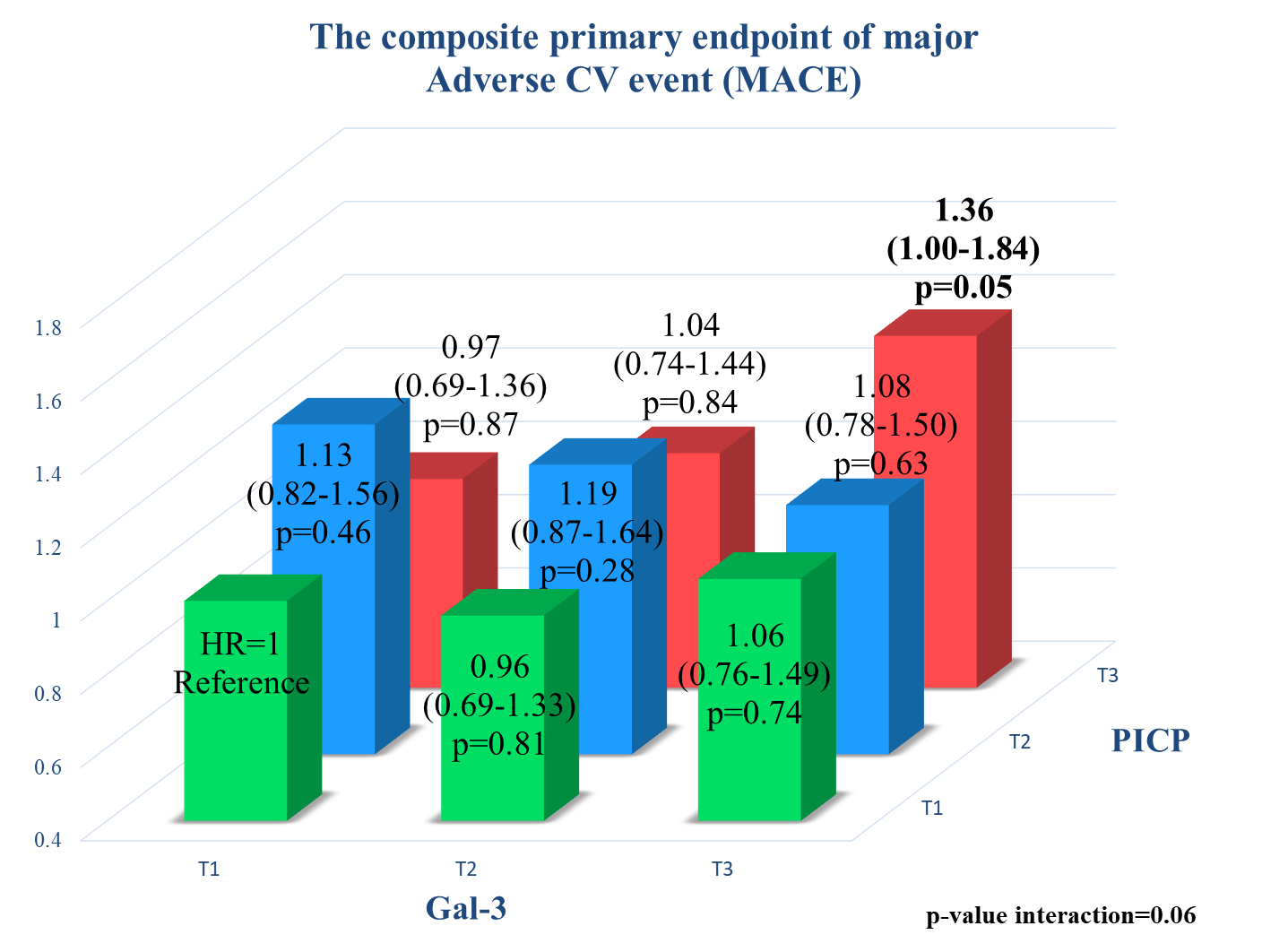

Supplement: Supplementary file 1 — Supplementary file1 (DOCX 3014 KB) [file 392_2021_1898_MOESM1_ESM.docx]
